# Beyond Heat Stress: Intestinal Integrity Disruption and Mechanism-Based Intervention Strategies

**DOI:** 10.3390/nu12030734

**Published:** 2020-03-11

**Authors:** Puqiao Lian, Saskia Braber, Johan Garssen, Harry J. Wichers, Gert Folkerts, Johanna Fink-Gremmels, Soheil Varasteh

**Affiliations:** 1 Division of Pharmacology, Utrecht Institute for Pharmaceutical Sciences (UIPS), Faculty of Science, Utrecht University, 3584 CG Utrecht, The Netherlands; p.lian@uu.nl (P.L.);; 2 Danone Nutricia Research, 3584 CT Utrecht, The Netherlands; 3 Food & Biobased Research, Wageningen University and Research, 6708 WE Wageningen, The Netherlands; 4 Institute for Risk Assessment Sciences (IRAS), Faculty of Veterinary Medicine, Utrecht University, 3584 CL Utrecht, The Netherlands

**Keywords:** heat stress (HS), intestinal integrity, nutritional supplements, resilience pathways, reactive oxygen species (ROS)

## Abstract

The current climate changes have increased the prevalence and intensity of heat stress (HS) conditions. One of the initial consequences of HS is the impairment of the intestinal epithelial barrier integrity due to hyperthermia and hypoxia following blood repartition, which often results in a leaky gut followed by penetration and transfer of luminal antigens, endotoxins, and pathogenic bacteria. Under extreme conditions, HS may culminate in the onset of “heat stroke”, a potential lethal condition if remaining untreated. HS-induced alterations of the gastrointestinal epithelium, which is associated with a leaky gut, are due to cellular oxidative stress, disruption of intestinal integrity, and increased production of pro-inflammatory cytokines. This review summarizes the possible resilience mechanisms based on in vitro and in vivo data and the potential interventions with a group of nutritional supplements, which may increase the resilience to HS-induced intestinal integrity disruption and maintain intestinal homeostasis.

## 1. Introduction

**Figure 1:**
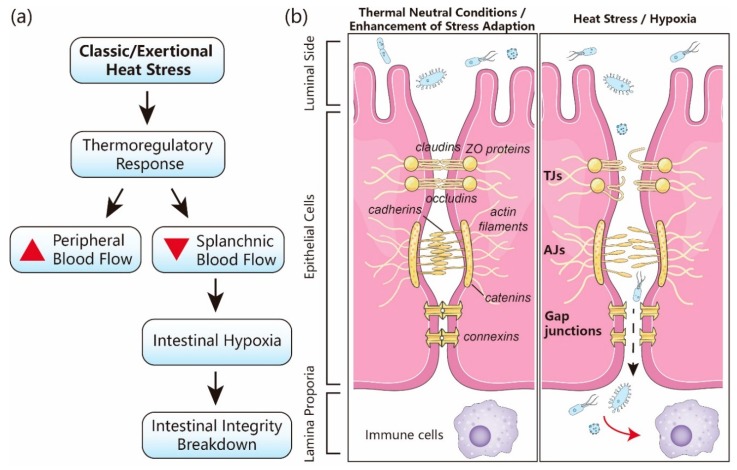
The sequence of events leading to heat stress-induced intestinal barrier damage. Hyperthermia induced by environmental or exertional heat stress (HS) stimulates the thermoregulatory mechanisms. (**a**) In the whole body, the thermoregulatory response shifts the splanchnic blood flow to the peripheral blood circulation, resulting in hypoxia in intestines and intestinal barrier dysfunction. (**b**) At the cellular level, hyperthermia leads to disruption of intestinal epithelial integrity, mainly by affecting the tight junctions (TJs) and adherens junctions (AJs), which are responsible for sealing the paracellular space between adjacent cells. Damage to TJs and AJs facilitates the transfer of luminal toxins and pathogens (light blue bodies) through the epithelial barrier into the lamina propria, harboring numerous immune cells that are activated and contribute to the exaggeration of the inflammatory reactions, which may further worsen the intestinal damage. ZO: zonula occludens protein.

The gastrointestinal (GI) tract is the largest surface of the body that is in contact with the outside environment. The intestinal epithelium is regarded as a physical and biochemical barrier between the luminal commensal and pathogenic microbial communities and the mucosal immune system [1]. Dysfunction of this barrier is caused by various pathological, toxicological, and physical stressors, including heat stress (HS), leading to local or systemic inflammatory reactions. Severe intestinal epithelial damage is considered as a major factor involved in HS-associated mortality [2,3,4,5,6]. The GI tract is affected by HS due to the thermoregulatory mechanism of the body shifting visceral blood flow towards the peripheral circulation to facilitate heat dissipation. This leads to visceral ischemia, followed by hypoxia, in visceral organs such as the intestines. Recent investigations unraveled the susceptibility of different organs to high body temperatures, demonstrating that the observed multi-organ failure is induced by a combination of heat-induced cytotoxicity, coagulopathies, and a systemic inflammation that affects not only the GI tract, but also other key organs and tissues, including the central nervous system [7], the kidneys [8], the liver [9], and the muscle tissue [10]. HS-induced hypoxic conditions in the intestine result in disturbance of the balance between the production of reactive oxygen species (ROS) and the antioxidant defense system, leading to epithelial damage and an inflammatory response [11] (Figure 1). Intestinal hypoxia can also induce local inflammation via barrier-independent pathways, involving cellular acidification by glycolysis, activation of autophagy and protective innate immune responses elicited by hypoxia-inducible factor (HIF)-1α [12,13,14].

HS has also been reported to negatively impact on production animal physiology, in particular poultry [15]. Modern poultry genotypes allow rapid growth but is also linked to higher metabolic activity and reduced heat tolerance [16,17]. This increased susceptibility of chickens to HS leads to multiple pathophysiological alterations also seen in humans, for example, intestinal barrier disruption and inflammation, oxidative stress responses and microbiome changes [18,19]. Considering the relevance of HS-induced cellular oxidative stress, disruption of intestinal integrity and the local and systemic inflammatory responses in humans and animals (poultry), the main aim of this review is to discuss promising nutritional intervention strategies, which may increase HS tolerance and to discuss their mechanisms of action, possibly explaining their beneficial effects in maintaining and supporting the intestinal homeostasis.

## 2. Stress Adaptation Signaling Pathways

### 2.1. Heat Shock Response (HSR)

**Figure 2:**
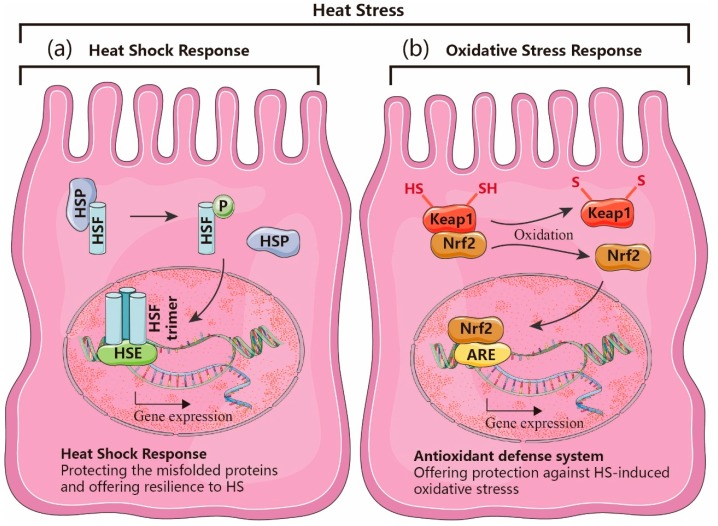
Schematic illustration of the heat stress (HS)-induced heat shock response and oxidative stress response. (**a**) Cells under HS conditions activate the heat shock response pathway, which is initiated by translocation and trimerization of heat shock factor-1 (HSF1) into the nucleus, where it binds to the regulatory heat shock elements (HSE) in the promoter regions of heat shock protein (HSP) genes. (**b**) Oxidative stress induced by HS results in the liberation of nuclear factor erythroid 2 related factor 2 (Nrf2) from Kelch-like ECH-associated protein 1 (Keap1) and the translocation of Nrf2 into the nucleus where it binds to the antioxidant response element (ARE) in the promotor region of antioxidant target genes, driving their expression.

The heat shock response was initially described as a specific molecular response of cells to adapt to elevated temperature. Later, various environmental and pathophysiological stressors, which cause protein aggregation or misfolding, were found to induce a similar reaction [20]. HSR is regulated by the activation of a family of interacting transcription factors, the so-called “heat shock factors (HSF)”, of which HSF1 is the best-characterized factor that is essential for the HSR [20]. Under physiological conditions, HSF1 is bound to heat shock proteins (HSP) in a monomeric form. Upon activation by cellular stressors, this complex of HSF1 and HSP dissociates and leads to trimerization and translocation of HSF1 into the nucleus and initiates the transcription of more HSP (Figure 2a). HSP fulfil an important role in binding to and protecting misfolded cellular proteins, a typical sign of HS. Among the variety of HSP family members [21], HSP70 is considered as the most stress-responsive protein, which is usually expressed at low basal levels and increases in response to stressors to protect the cells from proteotoxic damages by binding to damaged proteins and contributing to the refolding of unfolded or misfolded proteins. Subsequently, HSP inhibit apoptosis and even more important, the inflammatory response [20,22,23].

### 2.2. HSR and Oxidative Stress Response

Cell survival largely depends on the balance between ROS and cellular antioxidant mechanisms. The high reactivity of ROS can modify several cellular macromolecules, such as nucleic acids, proteins, and lipids [23]. HS is a potent inducer of ROS production, which leads to tissue damages as soon as the cellular redox defense system, consisting of glutathione (GSH), glutathione peroxidase, superoxide dismutase (SOD), and haem oxygenase 1 (HO-1), is exhausted [24]. Hyperthermia can provoke ROS production by adversely affecting mitochondrial membrane integrity and their electron transport chains [25,26], but is also able to hamper the antioxidant defense system directly [27]. The expression of the antioxidant system is mainly regulated by nuclear factor erythroid 2 related factor 2 (Nrf2), which is repressed in the cytoplasm by the regulatory protein Kelch-like ECH-associated protein 1 (Keap1) under physiological conditions. Dissociation of Nrf2 from Keap-1 upon oxidative stress, leads to translocation of Nrf2 to the nucleus where it binds to the ARE to induce the transcription of antioxidant proteins improving cell survival under stress conditions (Figure 2b) [28,29].

Local hypoxia caused by HS-induced changes in blood flow (from visceral to peripheral circulation) is identified as another major cause of ROS production. The main cellular response to hypoxia is triggered by HIF. Under normal conditions, the α subunit of HIF is rapidly hydroxylated by oxygen-sensitive prolyl hydroxylases (PHD) and then degraded in the proteasome [30]. However, under hypoxic conditions, PHD activity is inhibited, leading to HIF-1α stabilization and transcriptional activity so that cells can adapt to the hypoxic stress. One consequence of hypoxic signaling is the abnormal accumulation of ROS by complex III of the mitochondrial electron transport chain [31]. ROS generated under hypoxic conditions in turn contribute to HIF-1α stabilization [32] and activate the oxidative stress response via Nrf2, as previously discussed.

### 2.3. Resilience Pathways and Intestinal Barrier Integrity

Expression of HSP, in particular HSP70, is associated with the stabilization of the actin cytoskeleton of intestinal cells, preventing their aggregation under stress conditions [33]. Elevated levels of HSF1 and HSP70 are crucial in increasing the expression of actin fibers in epithelial cells of the GI tract. Upon activation under HS conditions, HSF1 binds to the occludin promoter region mediating the upregulation of the expression and improving the participation of occludin in junctional complexes [34]. Exogenous HSP70 added to cell cultures prevents HS-induced alteration in permeability. We recently showed that one of the possible mechanisms by which the antioxidant α-lipoic acid (ALA) and the amino acid arginine preserve the intestinal integrity under HS conditions could be related to the enhancement of HSP70 expression [35]. A possible mechanism by which HSP70 attenuates the epithelial barrier dysfunction under stress conditions will be through preventing the activation of conventional protein kinase C (cPKC), thereby reducing the myosin light chain (MLC) protein phosphorylation of the actin cytoskeleton [36,37]. Another member of the HSP family, the Apg-2 (a member of the HSP110 subfamily), interacts directly with zonula occludens protein-1 (ZO-1), regulating the transcriptional activity of ZO-1-associated nucleic acid binding protein [38]. The upregulation of HSP70 in Caco-2 cells (human epithelial colorectal adenocarcinoma cell line) following exposure to gliadin is associated with a redistribution of HSP70 towards the cytoskeleton, which improves the action of HSP70 in the maintenance of intestinal barrier function by allowing direct interaction with junctional proteins such as ezrin and E-cadherin [39].

Nrf2-Keap1 regulation is also linked with preserving intestinal barrier integrity. Jin et al. [40] observed higher intestinal permeability and plasma levels of endotoxin in the Nrf2-/- mice compared with wild-type mice in a traumatic brain injury-induced intestinal mucosa damage model. The upregulation of AhR-Nrf2 pathway and its target gene HO-1 (also known as HSP32) expression enhances the barrier function in the mice with inflammatory bowel diseases (IBD) [41]. Moreover, the extracellular signal-regulated kinase (ERK)/Nrf2/HO-1 signaling pathway can prevent the intestinal barrier damage by mediating mitophagy and increasing the expression of tight junctions (TJ) proteins under hypoxic conditions [42]. Interestingly, Nrf2 has two binding sites on the upstream of claudin-4 DNA sequence in the esophageal epithelium, highlighting the importance of Nrf2 in the TJ regulation [43].

### 2.4. Resilience Pathways and Immune System

Translocation of xenobiotics and bacterial products, following intestinal epithelial damage under HS conditions, may evoke an inflammatory response, which results in exaggeration of intestinal barrier dysfunction [36]. The anti-inflammatory properties of HSP70 have been studied extensively in chronic inflammatory disorders, such as IBD and celiac disease, as well as under conditions of hyperthermia [44,45,46]. The upregulation of HSP70 in response to HS is involved in the inhibition of pro-inflammatory cytokine expression [47]. HSP block the production of pro-inflammatory cytokines by inhibiting the translocation of Nuclear Factor-κB (NF-κB) to the nucleus [46]. Van Eden [48] reviewed the effect of HSP on expansion of anti-inflammatory regulatory T cells (T_reg_) and concluded that the introduction of HSP inducers into the diet can be considered as a therapeutic approach against inflammatory disorders. This specificity of HSP is not limited to endogenous (self) HSP, since administration of bacterial HSP is also an effective strategy in treatment of immune-challenging disorders [49,50,51]. Anti-inflammatory mechanisms of HSP are beyond the scope of this review and a more complete description of these mechanisms is presented in different review articles [52,53].

The crosstalk between ROS and Nrf2 and/or NF-κB, which activates the inflammatory cascade, is very complex and not yet fully elucidated [54]. Nrf2 and HSF1 regulate overlapping target genes and may compensate for each other [55]. HO-1 is considered as the most important Nrf2 target gene in facilitating NF-κB inhibition, which can be regulated by HSF1 as well. Additionally, exposure of HSF1 mutant cells to HS stimulates (although with delay) the upregulation of HSP70 and HO-1, which is mediated by Nrf2 [56]. 

Nrf2 also regulates intestinal immune function by affecting T cell polarization. The activation of Nrf2 inhibits the secretion of the Th1 cytokine IFNγ and interleukin (IL)-2 in early events, thereafter promoting CD4+ Th2 differentiation [57,58]. Moreover, Keap1 itself seems to be involved as a positive regulator of NF-κB in inflammatory signaling [59].

In conclusion, activation of both the heat shock and oxidative stress responses contribute to an increased resilience to heat stress conditions and may help to mitigate the stressful effects of increased body temperature and decreased oxygen accessibility by regulating the expression of protective proteins, such as HSP, Nrf2, and HO-1. HSP, Nrf2 and HO-1 in the intestinal epithelia can interact with junctional complexes and components of the immune system resulting in the restoration of local homeostasis following hyperthermia and hypoxia.

## 3. Intervention Strategies against HS

As mentioned earlier, hyperthermia and subsequent hypoxia not only provoke ROS production, but also directly hamper the antioxidant defence system [27].

Independent from these direct effects on cells of the intestinal barrier system, the gut microbiota is a common target of HS conditions [60,61]. Alterations in the composition of the gut microbiota, together with the HS-induced impairment of the barrier function, increase the likelihood of opportunistic intestinal infections. In turn, pre- and probiotics have gained recent interest, as they are able to stabilize the intestinal microbiota under stress conditions. A variety of antioxidant substances, fatty acids, and selected amino acids are also commonly recommended to mitigate disease conditions closely associated with cellular oxidative stress, as will be discussed below.

### 3.1. Microbiota Modulation

The gut microbiota, which comprises a vast array of microorganisms, has a key effect on the regulation of host nutrition and metabolism, as well as on the stimulation of gut maturation, development, proliferation, and immune homeostasis [60,62]. A variety of host conditions, including diet, immune reactions, infections, and usage of antibiotics influences the gut microbiota. Stress conditions, including HS, induce alterations in the microbiota balance, which may result in the colonization of enteric pathogens [63], and intestinal inflammatory responses [64]. Stabilization of the gut microbiota composition by pro-, pre-, syn-, and postbiotics is considered as an effective strategy to improve gut health and to protect the intestines against stress conditions [65,66,67,68].

#### 3.1.1. Probiotics

Probiotic bacteria are defined as “living microorganisms which exert health promoting benefits when administered in adequate amounts” [69]. A large range of bacteria are considered as probiotics, while the most common strains belong to *Lactobacillus* and *Bifidobacterium* species [70]. While probiotics were initially identified based on the competitive displacement of pathogens, they have also shown to be protective against non-infective disorders, such as dextran sodium sulfate (DSS)-induced colitis in mice, and influence the morphology and the immunological homeostasis in the GI tracts of animals and humans [70,71]. Furthermore, the beneficial effects of probiotics are related to the improvement of different components of the gut barrier system, including the regulation of immune reactions, and enhancement of intestinal epithelial cell integrity [72,73].

A clinical study showed that four weeks of daily supplementation with a probiotic mixture containing strains of *Lactobacillus*, *Bifidobacterium,* and *Streptococcus* species maintains the intestinal integrity and reduces the penetration of LPS into the blood in male runners affected by intense exercise-induced HS [70]. Furthermore, supplementation with a matrix and six probiotic strains (*B. bifidum*, *B. lactis*, *E. faecium*, *L. acidophilus*, *L. brevis,* and *L. lactis*) for 14 weeks reduced the concentration of zonulin in feces of athletes, and improved intestinal barrier integrity [74]. It is known that increased zonulin concentration in feces is related to enhanced gut permeability and changes in tight junction competency. Moreover, in vitro evidence demonstrated that a mixture of three different strains of *Lactobacillus* species increased trans-epithelial electrical resistance (TEER) values, occludin mRNA expression, and mucus production in Caco-2:HT29–MTX epithelial co-cultures [75]. Model experiments in broiler chickens indicate that probiotics successfully alleviate the detrimental effects of HS on the microstructures of the small intestine, such as reduced villus height and density [76,77]. An ex vivo study from Song et al. [77] showed that 42-day treatment with a *L. plantarum*, *B. licheniformis,* and *B. subtilis* mixture could restore the decreased trans-epithelial electrical resistance (TEER) levels and subsequently increased the paracellular permeability in the jejunal segment of HS-exposed chickens. The beneficial effects of this probiotic mixture was associated with an increase in occludin and ZO-1 protein expression [77]. 

*Bacilli* are also commonly used as human probiotics for their multi-bioactivity and high bio-safety [78]. Feed supplementation with *B. subtilis* for 42 days improves the intestinal integrity in chickens by increasing the expression of occludin, claudin-2, and claudin-3 in the jejunum and the ileum [79]. Similarly, pretreatment of *B. subtilis* for two days diminishes the intestinal morphological changes and bacterial translocation as well as lipopolysaccharides (LPS) penetration to the blood flow in rats exposed to HS [80]. 

Probiotics do not only interact with the bacterial populations in the intestine, but there is also an interplay between microbiota and the host’s defense system. For example, probiotics directly and/or indirectly modulate different signaling pathways that regulate the intestinal integrity, including Rho family GTPases, protein kinase C (PKC), and mitogen-activated protein kinase (MAPK). The protective effect of a Gram-negative *E. coli* Nissle probiotic on intestinal integrity of T84 cells (colonic adenocarcinoma epithelial cells) challenged by enteropathogenic *E. coli*, is related to the stabilization of ζ isotype of protein kinase C (PKCζ), thereby preventing the phosphorylation and dissociation of ZO-2 from the TJ network [81]. In agreement with these findings, the epithelial barrier function in T84 cells is enhanced by the four strains of Gram-positive probiotic *Lactobacillus* species via their effect on adherens junctions (AJs), including E-cadherin and β-catenin, by reducing the abundance of δ isotype of protein kinase C (PKCδ) in membrane junctional complexes [82], highlighting the notion that probiotics with different Gram-staining status target distinct signaling pathways regulating different intercellular junctions. *L. brevis* produces a bioactive molecule, polyphosphate, through activation of the integrin–p38 MAPK pathway, which leads to increased HSP expression at protein level and prevention of oxidant-induced intestinal barrier disruption [83]. In addition, the protective effects of the probiotic strains of *S. thermophiles* and *L. acidophilus* on occludin phosphorylation in human intestinal epithelial cells challenged with enteroinvasive *E. coli*, can be inhibited by a Rho kinase inhibitor [84].

The probiotic *B. licheniformis* supports the gut mucosal immunity in broiler chickens exposed to HS, by preventing HS-induced increase in pro-inflammatory cytokines and decrease in intraepithelial lymphocytes, the IgA secreting plasma cells and mucin production [85]. *B. subtilis* B10 stimulates the mucosal immunity development in broiler chickens by increasing IgA secretion and mRNA expression of the anti-inflammatory cytokine IL-10 [79]. Furthermore, clinical studies showed that dietary supplementation with a probiotic mixture increases the post-exercise plasma concentrations of IL-10 in exercise-induced HS [70]. 

The immune-regulatory properties of probiotics have been studied extensively in treatment of diseases affecting the intestinal mucosal immunity, such as IBD [86,87]. It seems that the mechanism by which probiotics exert anti-inflammatory properties, is through inhibition of NF-κB [88]. Moreover, probiotics stimulate CD103+ dendritic cells to produce IL-10 and IL-27 via the toll-like receptors (TLR)-2/MyD88 pathway [89].

Overall, probiotics modulate both the innate system (via natural killer cells, dendritic cells, macrophages, epithelial cells, and granulocytes) and the adaptive system (Th1, Th2, Th17, T_reg_, Tc, and B cells) [90,91]. The activation of an innate immune response by probiotics is mainly facilitated by microbe-associated molecular patterns, including bacterial cell wall polysaccharides and peptidoglycan [92], which interact with TLR, C-type lectin receptors, and nucleotide oligomerization domain-like receptors [93]. However, it should be taken into account that as yet no single probiotic is found to exert all the above-mentioned effects.

#### 3.1.2. Prebiotics

Dietary prebiotics are described as “selectively fermented ingredients that result in specific changes in the composition and/or activity of the GI microbiota, thus conferring benefit(s) upon host health” [94]. Human milk oligosaccharides (HMO), a major component of colostrum, represent the first prebiotic in the human diet. A recent study confirmed the protective action of HMO by supplementing neonatal mice formula and media for Caco-2 cells, and found an improved response against hypoxia-induced injuries [95]. Various attempts have been made to design alternative prebiotic oligosaccharides that mimic the health promoting effects of HMO, including galacto-oligosaccharides (GOS) and fructo-oligosaccharides (FOS), which are widely used in infant formulas [96]. These non-digestible oligosaccharides are not hydrolyzed by mammalian digestive enzymes and reach the distal intestines, where they modify the autochthonous microbiota and exert a beneficial effect on the gut microbiota [97]. Selective stimulation of beneficial bacteria, such as *Lactobacillus* and *Bifidobacterium* species, can induce immunomodulatory effects, enhance the intestinal integrity and preserve the intestinal micro-structures [98,99]. The gut microbiome targets different intracellular pathways via fermentation of non-digestible oligosaccharides and the subsequent production of short chain fatty acids (SCFA), such as acetate, propionate, or butyrate [100]. The postbiotic, butyrate, increases the antioxidant glutathione and decreases ROS production when applied directly to the human colon cells [101,102], which would probably modulate the HS-induced intestinal damage by ROS [103]. Moreover, SCFA, such as propionate and butyrate, activate free fatty acid receptor (FFAR) 2 [also known as G-protein-coupled receptor (GPR) 43] and FFAR3 (GPR41) to stimulate the mucus secretion and facilitate the production of anti-inflammatory cytokine IL-10 [104,105].

In vivo investigations in chickens exposed to HS have shown that supplementation of the diet with mannan-oligosaccharides (MOS) and cello-oligosaccharides (COS) mitigated the heat-induced changes in intestinal morphology and intestinal barrier function [106,107]. Furthermore, MOS enhance the intestinal integrity by increasing villus height, the number of goblet cells, and the populations of *lactobacilli* and *bifidobacteria*, while at the same time reducing the *E. coli* load in the ceca of chickens [108]. HMO incubated with *B. longum infantis* increased IL-10 expression and ZO-1, occludin and junctional adhesion molecule (JAM)-A mRNA transcription in Caco-2 and HT-29 cells [109]. In turn, an in vitro experiment reported that FOS improved the viability and heat tolerance capacity of two strains of *lactobacillus* species: *L. plantarum* and *L. acidophilus* [110]. Chitosan oligosaccharides attenuate inflammatory infiltrates and epithelial degeneration in mice colon, and increase TEER in T84 cells [111]. Our group showed that dietary GOS supplementation diminishes the disruption of intestinal integrity by preventing the alterations in TJs and AJs in the jejunum of broiler chickens exposed to HS [112]. In addition, GOS increase the number of intestinal *bifidobacteria* in rats and play a key role in prevention of intestinal integrity disruption by increasing the mRNA and protein expression of occludin [113]. 

Besides the effects on the gut microbiota, microbiota-independent effects and direct interactions of these oligosaccharides with different (immune) cells have raised more attention in recent years [114]. FOS was found to directly promote barrier integrity by increasing ZO-1 and occludin expression, through a protein kinase C (PKC) δ-dependent mechanism, in pathogen-challenged Caco-2Bbe1 cells (a Caco-2 subclone) and human intestinal organoids [115]. Our in vitro investigations highlighted the effect of GOS on direct regulation of the intestinal integrity and junctional complexes to prevent the disruption of intestinal integrity induced by HS [116]. Moreover, pre-treatment with GOS prevents the disruption of intestinal integrity by accelerating TJ reassembly and stabilizing the expression and cellular distribution of claudin-3 TJ protein in Caco-2 cells [117]. The microbiota-independent effect of non-digestible oligosaccharides on intestinal epithelial integrity depends on the oligosaccharide structure, size, and concentration [118]. Although further research is needed to unravel the exact mechanism involved in the direct regulation of intestinal integrity by oligosaccharides, an in vitro study with T84 cells showed that chitosan oligosaccharides promote TJ assembly by activating 5' adenosine monophosphate-activated protein kinase (AMPK) through calcium-sensing receptor-phospholipase C-IP3 receptor channel-mediated calcium release [119]. 

We showed in broiler chickens that dietary GOS prevents the HS-induced mRNA upregulation of IL-6 and IL-8 in the jejunum. This effect could be related to the GOS-preserved intestinal integrity [112]. In addition, GOS prevent the HS-induced TLR-4 upregulation in the jejunum [112]. Disruption of intestinal integrity followed by translocation of luminal antigens and pathogens through the intestinal epithelium exaggerates TLR signaling, facilitates immune responses, and eventually leads to the development of intestinal inflammation and intestinal injury [120,121,122]. Another study from Wang et al. also showed that by reducing the expression of TLR-4 and NF-κB, and accelerating the turnover of crypt cells, HMO protect intestinal epithelial cells from necrotizing enterocolitis (NCE) injury in mice [123]. Additionally, TLR-4 is described as a stress-related biosensor in the initial injury responses [124] and may contribute to the intestinal barrier disruption, since it is demonstrated that TLR-4 knockout mice are protected from HS-induced intestinal hyper-permeability and microvascular endothelial barrier dysfunction [121,125]. 

In recent years, the immune-regulatory effects of prebiotics to prevent intestinal disorders, such as IBD and NCE, (food) allergy, or intestinal damage related to mycotoxin exposure are extensively studied [111,126,127,128,129,130,131,132,133]. GOS suppress the mycotoxin-induced increase in CXCL8 in Caco-2 cells as well as the murine CXCL8 analogues (CXCL1 and CXCL2) in the intestine [117]. Moreover, dietary GOS mitigate the inflammation-induced expression of the alarmin IL-33 in two different murine models [134]. Jeurink et al. [98] reviewed the different mechanisms which can underlie the immune effects of dietary oligosaccharides.

In summary, pro- and pre-biotics exert their therapeutic and prophylactic effects on HS-induced intestinal integrity disruption by modulating immune function, improving gut barrier integrity by stimulating mucus production and modulating junctional proteins, increasing antioxidative capacity, and supporting the resident microbiota. The effects of pro- and pre-biotics on intestinal integrity and immunomodulation are summarized in Table 1.

### 3.2. Antioxidants

Preserving the redox balance, by the participation of Nrf2 and Keap1, is crucial to control the overproduction of ROS and maintain intestinal integrity under HS conditions [138]. 

ROS are signaling molecules in physiological levels but are also responsible, at a high concentration, for intracellular damage [139]. Under physiological ROS production levels, proteinaceous antioxidants act as a defence mechanism to neutralize ROS production [140]. However, conditions such as HS are associated with excessive ROS generation [141,142]. Therefore, supplementation with oral exogenous antioxidant components or pro-oxidants, which can beneficially trigger the Nrf2-Keap1 pathway, may help to alleviate oxidative stress and its contribution to the pathogenesis of HS in the intestine.

#### 3.2.1. α-Lipoic Acid (ALA), a Fatty Acid with Antioxidant Properties

ALA is synthesized from octanoic acid in the mitochondria and is present in pro- and eukaryotic cells, being identified as a potent cellular antioxidant. Both reduced and oxidized forms of ALA retain the antioxidant potency by scavenging free radicals, exhibiting metal chelating activity, and through their involvement in redox regeneration of other antioxidants (vitamin C and E) [143,144]. ALA is used as treatment for diverse pathologies associated with redox imbalances, including diabetes, ischemia-reperfusion injury, and heavy metal poisoning. However, ALA may act as mild pro-oxidant by slightly increasing ROS concentrations to activate Nrf2 and HSF, and therefore increasing the resilience to stress conditions [144]. In addition to redox-regulating effects, ALA may enhance gut integrity and exert anti-inflammatory properties [145,146,147]. ALA hampers the disruption of intestinal integrity and modulates the intestinal inflammation in models of HS, post-weaning diarrhea and ulcerative colitis [145,146,147].

Although the intestinal integrity-associated effects of ALA are not extensively studied under HS conditions, investigations in Caco-2 cell monolayers exposed to HS showed that ALA prevents the disruption of intestinal integrity by maintaining protein expression and distribution of the AJ, E-cadherin [147]. Furthermore, ALA stimulates proliferation of intestinal epithelial monolayers and facilitates the reassembly of TJs [147]. 

ALA supplementation preserves the intestinal integrity in oxidative and inflammatory disorders associated with intestinal damage [145,146,148]. ALA stimulates the recovery of the intestinal epithelial architecture by increasing transcription and translation of occludin and ZO-1 TJ proteins in a rat model for post-weaning diarrhea. These findings are confirmed by in vitro studies with IEC-6 intestinal epithelial cells [146]. ALA mitigates the intestinal morphological damage by preventing the decrease in villus height and increase in crypt depth in glycinin-induced anaphylactic reactions in rats [148]. Additionally, ALA co- and post-treatment decreases ulcerative colitis-induced gut permeability by maintaining the expression of occludin in mice [145]. These effects are, at least in part, related to the regulation of the redox balance since oxidative stress induces a tyrosine-kinase-dependent dissociation of E-cadherin-β-catenin and occludin-ZO1 complexes, which leads to their cellular redistribution and a loss of barrier integrity [149]. Additionally, the pro-oxidant activity of ALA stimulates the transcriptional activity of HSF1 to induce the expression of HSP70. HSP70 may be involved in the maintenance of barrier integrity through direct interaction with TJ proteins and stabilizing the junctional complexes [39]. 

The anti-inflammatory effects of ALA are closely related to its antioxidant properties. As mentioned before, activation of the NRF2 transcription factor by ALA results in the induction of HO-1, which exerts anti-inflammatory effects by degrading intracellular haem to free ion, carbon monoxide and biliverdin [150,151]. In the last decade, the effect of ALA in the transcriptional regulation of genes associated with inflammatory pathways were highlighted [144,152,153]. Exposure of Caco-2 cells to HS increases cyclooxygenase-2 (COX-2) mRNA expression, the inducible COX, which is attenuated by ALA pre-incubation [147]. COX-2 catalyses the rate-limiting step in the conversion of arachidonic acid into inflammatory prostaglandins. Interestingly, inhibition of COX-2 by ALA is speculated to be important in the prevention of ulcerative colitis in rats [154]. ALA, co- and post-treatment in mice with ulcerative colitis, not only prevents the transcription of COX-2, but also significantly reduces various inflammatory markers, such as myeloperoxidase, IL-17, IL-6, and TNF-α in the colon [145]. 

These findings support the hypothesis that the anti-inflammatory and protective effects of ALA under stress conditions are mainly attributable to the inhibition of IκB/NF-κB phosphorylation, hence preventing the activation of NF-κB [153]. 

#### 3.2.2. Resveratrol, a Plant Polyphenol Compound

The polyphenolic compound resveratrol (3,5,4′-trihydroxytrans-stilbene) is present in grape skin, grape seeds, and peanuts. Resveratrol is an important bioactive compound, which displays a strong antioxidant and anti-inflammatory capacity. The significance of resveratrol in ameliorating the deleterious effects of HS has been reviewed previously [155]. Indeed, enhancing resilience to oxidative stress via administration of antioxidants or pro-oxidants (compounds which moderately induce the ROS just enough to activate the antioxidant defence system) has been introduced as an effective strategy in preventing HS-induced gut-associated dysfunction.

Although the exact underlying mechanism between the antioxidant capacity of resveratrol and the protective effect on gut homeostasis is not fully understood, the induction of HSP, in particular HSP70, HSP90, and HO-1, is one of the major effects of resveratrol to preserve cellular homeostasis under stress conditions [156,157,158]. Resveratrol-induced HSP70 expression reduces the temperature threshold of the heat-shock response and preconditions the cells to cope with more severe or lethal stress levels [159]. Resveratrol reduces glutathione disulphide (GSSG) formation, maintaining GSH in its reduced form to prevent ROS-induced cellular damage [160] and, in addition, inhibits the H_2_O_2_-induced lipid peroxidation by reducing malondialdehyde (MDA) formation, while increasing SOD activity and inhibiting the elevated intracellular expression of ROS in Caco-2 cells [161]. Resveratrol-induced HO-1 signaling is crucial in common expression of TJ proteins by inhibiting the PKC activity and P38 phosphorylation [161]. 

Resveratrol alleviates the HS-induced intestinal damage by preserving villus height to crypt depth ratio in chickens [162]. In vitro investigations using IPEC-J2 intestinal epithelial cells showed that resveratrol ameliorates intestinal epithelial integrity breakdown by increasing TEER values, reducing bacterial translocation, and decreasing the paracellular permeability. These effects were mainly associated by enhancement of claudin-4 TJ protein assembly [163]. Moreover, in vitro and in vivo studies indicated that pre-treatment with resveratrol significantly hampered H_2_O_2_-induced damage to occludin and ZO-1 proteins in a concentration- and time-dependent manner in Caco-2 cells by upregulating HO-1 expression. In models of intestinal integrity disruption in rats, resveratrol treatment attenuated the gut hyperpermeability resulting from oxidative stress [161]. Recent investigations by Mayangsari and Suzuki [164] pointed out that resveratrol ameliorated DSS-induced ZO-1, ZO-2, occludin, JAM-A, claudin-2, claudin-3, claudin-4, and claudin-7 suppression.

Oxy-resveratrol, an isomer of hydroxylated resveratrol, effectively decreases fluorescein isothiocyanate (FITC)-Dextran transport through the Caco-2 monolayer in a concentration-dependent manner [165]. Occludin, ZO-1 and claudin-1 expression was significantly increased in oxy-resveratrol-treated Caco-2 cells compared to untreated cells, which might improve TJ integrity via PKC and MAPK-mediated pathways.

Besides preserving the intestinal integrity, the induction of HSP by resveratrol induces immune-regulatory effects, since HSP are activators of anti-inflammatory regulatory T cells and HSP induction blocks the NF-κB activation by stabilizing IκB-α [166]. Resveratrol treatment prevents the HS-induced transcription of NF-κB [155,162]. In turn, resveratrol exerts an anti-inflammatory capacity by inhibiting COX-2 expression. COX-2 is a heat-responsive gene, upregulated as a HSF1 target gene under HS conditions [167,168]. In addition, resveratrol reduces the transcription of pro-inflammatory cytokines, such as IL-6 and IL-1β, as well as COX-1 in Caco-2 cells, induced by LPS in combination with a cytokine cocktail [169]. This underlines their safe use as preventative anti-inflammatory agent. As importantly, the immune-related cellular mechanisms modulated by resveratrol are associated with the stress-activated protein kinases/c-Jun N-terminal kinase (SAPK/JNK), ERK 1/2, p38 MAPK and spleen tyrosine kinase (Syk) signaling pathways [164].

In conclusion, to restore the imbalance of the antioxidative system, quenching of excessive ROS by antioxidants, such as α-lipoic acid and resveratrol, is key. This process involves the improvement of the antioxidative enzyme system, activation of oxidative stress-modulating proteins, and inhibition of the inflammatory response. The effects of α-lipoic acid and resveratrol on intestinal integrity and immunomodulation are summarized in Table 2.

### 3.3. Polyunsaturated Fatty Acids (PUFA)

Polyunsaturated fatty acids eicosapentaenoic acid (EPA) and docosahexaenoic acid (DHA) are members of the omega-3 fatty acid family. In the human diet, these fatty acids are mainly derived from fish and fish oil with high fat content. The importance of adequate EPA and DHA intake for the development of the fetal nervous system has been demonstrated [170]. The effects of fatty acid intake on chronic diseases, including obesity, diabetes, cancer, arthritis, asthma, and cardiovascular diseases, are widely recognized [171]. In addition, low doses of PUFA exert antioxidant activities [172] and are cellular antioxidants that might positively modulate important physiological functions such as antioxidant capacity and enzymatic activities [173]. The modulation mechanism involves quenching of intracellular ROS generation and direct inhibition of Nox4 [172]. Low dose EPA/DHA-fed rats displayed a higher activity of the antioxidant machinery, including an enhanced SOD and catalase (CAT) activity in addition to a reduction in total nitrate/nitrite content [174].

EPA effectively attenuated the decrease in TEER and impairment of intestinal permeability in HRP flux induced by heat exposure [175]. EPA significantly elevated the expression of occludin and ZO-1 in CaCo-2 cells. The distortion and redistribution of TJ proteins, and disruption of morphology were also effectively prevented by pre-treatment with EPA [175]. A significant reduction in mucosal damage in the gut of rats was observed after an EPA/DHA supplemented diet, as reflected by the maintenance of total protein content [174]. In Caco-2 cells, upon an acute inflammatory stimulus, DHA partially restored the occludin intensity in tight junction complexes, and preserved the ZO-1 localization and function by increasing TEER values and decreasing Lucifer Yellow (LY) flux in a concentration-dependent manner [176]. DHA and EPA counteracted chronic stress-induced dysfunctions, such as the downregulation in ZO-1, occluding, and E-cadherin, and aberrant microbiota composition and their metabolites, mainly acetic acid, propionic acid, and butyric acid [177].

DHA and EPA have anti-inflammatory activities and are used as “immunonutrients” [178]. DHA and EPA decreased pro-inflammatory cytokines interferon (IFN)-γ, TNF-α, IL-1β, and IL-6 production in the intestine of mice exposed to chronic stress [177]. This effect was mediated by up-regulating GPR120 and down-regulating TAK1/NF-κB p65 signaling. Zhao et al. [179] reported that in colonic mucosa, DHA pre-treatment decreased immune cell infiltration, down-regulated IL-17, TNF-α, and INF-γ levels, and improved intestinal epithelial barrier function. In human colon cancer HT-29 cells, DHA and EPA inhibited ERK-1 and -2 phosphorylation and HIF-1α protein over-expression by reducing COX-2 expression and prostaglandin (PGE)_2_ levels [180]. Furthermore, the other cellular mechanisms accounting for immunomodulatory effects also included down-regulation of inducible isoform of NOS (iNOS) and cyclic guanosine monophosphate (cGMP) [181]. In addition, EPA and DHA are able to increase cytokeratin 20 and mucin 2 gene and protein expression, which can enhance the intestinal immunological barrier by providing binding sites for antibacterial peptides [177,182].

In summary, PUFA exert protective effects on the intestinal epithelial cell monolayer by protecting the barrier function and by anti-inflammatory activity. However, PUFA seem to have distinct effects at different concentration ranges in different disease models and in different cell lines. More in vivo studies are needed to determine the precise beneficial effects of PUFA on (HS-induced) intestinal disorders. The effects of DHA and EPA on intestinal integrity and immunomodulation are summarized in Table 3.

### 3.4. Amino Acids

Nutrition deprivation experiments proved that amino acids are critical to the gut barrier and the gut microbiota [183,184]. In the last decade, awareness of the nutritional relevance of some amino acids has been broadened from nutritional-only to therapeutically-important agents, due to their ability to modify cell signaling and to modulate gut-associated disorders [185,186,187]. Functional amino acids, including arginine and glutamine enhance intestinal mucosal immunity, abolish the oxidative damage, trigger proliferation of enterocytes and enhance gut barrier function. Glutamine is not only the main fuel for enterocytes, but also plays a key role in mitogenesis, cytoprotection and barrier function [188,189]. In addition, arginine through activation of focal adhesion kinase (FAK), mammalian target of rapamycin (mTOR), as well as nitric oxide (NO) cascades, actively participates in wound healing, and mucosal repair in intestinal epithelium [190].

#### 3.4.1. Arginine

Arginine supplementation attenuates the adverse effects of heat stroke in in vivo models [191,192]. Therapeutic administration of arginine in mice and rats exposed to HS reduces the adverse effects of multi-organ failure, such as circulatory shock and cerebral ischemia, leading to improved survival [191,192,193]. Arginine supplementation is also involved in the maintenance of intestinal homeostasis. Oral administration of arginine significantly enhances the intestinal recovery and accelerates the mucosal repair following ischemia-reperfusion injury in rats [194]. In vitro, arginine suppresses apoptosis and cell death induced by LPS in porcine small intestinal cell line IPEC-1 cells [195]. However, arginine in intestinal inflammation is a “double-edged sword”, because supra-physiological concentrations of arginine (>10 mM) may inhibit cell migration in intestinal wound edges and play a deleterious role in the pathogenesis of inflammation [196,197]. Similarly, supra-physiological concentrations of arginine worsen the mucosal damage and gut barrier function after ischemia/reperfusion injury in rats [198]. However, arginine supplementation in a physiological range plays an important role in the metabolic synthesis pathways, like the polyamine and NO production, which are involved in multiple cellular signaling pathways in enterocytes, including intestinal protein synthesis, blood flow, healing processes and intestinal immunity [199,200].

HS blocks the physiological NO production [201], which, hence, will significantly increase the body heating rate, reduce the heat dissipation and increase the intestinal epithelial permeability [202,203]. Therefore, basal NO level is a key factor in the enhancement of resilience to stress conditions [204]. Physiological NO production enhances the tolerance to HS by reducing O_2_ costs under extensive exercise [205,206].

Dietary arginine supplementation is important in attenuating the intestinal integrity disruption caused by exertional hyperthermia [202]. Pre-treatment of Caco-2 cells with non-toxic arginine concentrations prevents the disruption of intestinal integrity [35]. Arginine supplementation improves the intestinal integrity and preservation of TJs in experimental models of IBD and hypoxia [207,208]. Arginine supplementation increases the mucus production as well as fluid secretion and inhibits intestinal hyper-motility in rats [209]. Additionally, in vitro and in vivo studies showed that supplementation with arginine prevents bacterial translocation by reducing intestinal necrosis, increasing villus height, and attenuating gut mucosal injury [200,210].

Different mechanisms may be involved in the arginine-induced tolerance of intestinal epithelial cells to HS, including:

1. The NO synthesis pathway: Arginine, as a precursor of NO production, stimulates the enzyme NO synthase (NOS) isoforms to facilitate the synthesis and bioavailability of NO [211]. The constitutive form of NOS (cNOS), which includes endothelial NOS (eNOS) and neuronal NOS (nNOS), generates relatively small amounts of NO, while iNOS produces a quantitatively larger amount of NO and is expressed in cells of the immune system as well as in intestinal epithelial cells [186,212,213].

Although the precise mechanisms through which NO protects intestinal integrity is not fully understood, NO regulates the intestinal integrity by modulating intracellular signaling pathways related to protein tyrosine phosphorylation in epithelial cells [214]. Protein tyrosine phosphorylation of TJ and AJ proteins, which can be induced by diverse oxidation-related stimuli including HS, is involved in barrier disruption under oxidative stress conditions [215,216]. Additionally, NO signaling plays a key role in intestinal re-epithelialization and maintenance of intestinal integrity following mucosal injury [208,217].

2. Mammalian target of rapamycin (mTOR) pathway: Maintaining the intestinal epithelial function by arginine can also be related to activation of the mTOR pathway [196,218]. Arginine induces the downstream mTOR pathway by phosphorylation and activation of the protein synthesis regulator 70-kDa ribosomal protein S6 kinase (p70^S6k^) [219]. Activation of p70^S6k^ by arginine increases protein synthesis, proliferation, and migration in disease conditions that induce intestinal epithelial injury [200]. For instance, oral administration of arginine in a porcine model of enteritis augments intestinal protein synthesis and attenuates intestinal permeability via mTOR signaling and p70^S6k^ activation [218].
3. Arginase pathway: Metabolism of arginine via the arginase pathway results in the production of ornithine and polyamine, which promote intestinal epithelial repair and restitution processes [196]. Polyamines are involved in the regulation of cell-cell interactions and E-cadherin expression, being critically important for the maintenance of intestinal epithelial integrity [220]. Additionally, polyamines are important stress-responsive molecules, which facilitate the activation of HSF1 to induce HSP expression [221,222].

NO synthesis from arginine and the subsequent production of intestinal secretory immunoglobulin A (sIgA), modulate the expression of Th1/Th2 cytokines and prevent the exaggerated inflammatory responses followed by intestinal damage [209,223]. One of the beneficial effects of arginine supplementation in preventing the intestinal inflammation in rats exposed to HS, may be mainly attributable to these immune-regulatory effects [202]. Arginine supplementation reduces the expression of pro-inflammatory cytokines in the colon of mice with an experimental sodium dextran sulphate-induced colitis [207].

It is likely that the main mechanism of action by which arginine modulates the inflammatory responses is the iNOS-induced NO production, which inhibits NF-κB [224], since inhibition of iNOS leads to the loss of all clinical benefits of arginine in the intestines [207].

#### 3.4.2. Glutamine

Nutritionally supplementary glutamine is traditionally classified as a non-essential amino acid. Glutamine is considered as an important precursor for the synthesis of nucleotides and proteins. Glutamine availability is effective in the maturation of rapidly proliferating intestinal epithelial cells in the gastrointestinal tract [225]. Glutamine is a critically important fuel for the intestinal epithelial cells and is essential for preserving the intestinal mucosal barrier in humans and animals [226,227]. In addition, circulating or luminal glutamine improves the gut function and mucosal integrity [228]. Glutamine is important in regulating many key metabolic processes, such as protein synthesis, regulation of cellular redox status, and immune responses [229,230,231]. One of the most described characteristics of glutamine is the enhancement of cell survival by inducing the expression of HSP [232,233,234].

Animal research and clinical studies revealed that insufficient intake of glutamine is associated with the development of intestinal diseases and mucosal barrier breakdown, which can be reversed by glutamine supplementation [235,236,237]. The effect of glutamine supplementation on gut micro-structures, such as amelioration of villus atrophy, has been previously described in different in vivo studies [238,239]. Glutamine is essential to preserve the intestinal epithelial integrity, since the depletion of glutamine leads to the loss of TJ proteins and increased intestinal paracellular permeability as observed in epithelial cells [240,241]. Incubation with L-glutamine significantly enhanced epithelial barrier function in primary porcine jejunal enterocytes by increasing occludin, claudin-4, JAM-A, ZO-1, ZO-2, ZO-3 protein expression [188]. The glutamine-induced upregulation of HSP70 in the intestine prevents intestinal mucosal injury by improving the intestinal antioxidant capacity, such as elevating the superoxide dismutase, glutathione peroxidase, and total antioxidant capacity inhibiting lipid peroxidation [242]. Interestingly, the glutamine-induced HSF-1 and HSP70 gene expressions are associated with a prevention of TJs disruption (ZO-1 and occludin), and thereby with protecting the intestinal epithelial cells from injuries caused by HS [243,244]. Another mechanism by which glutamine exerts protective effects against intestinal oxidative stress is related to the up-regulation of HO-1. Inhibition of HO-1 abolishes the preventive effect of glutamine against intestinal damage caused by radiation in colon epithelial cells [245]. Glutamine attenuates the disruption of intestinal epithelial tight junctions (ZO-1, claudin-1, occludin) and adherens junctions (E-cadherin and β-catenin) caused by acetaldehyde in Caco-2 cell monolayer [246]. Glutamine is possibly involved in the regulation of apical junction complexes via tyrosine phosphorylation of the epidermal growth factor (EGF) receptor, since the protective effect of glutamine was eliminated by a specific inhibitor of EGF receptor tyrosine kinase [246]. Glutamine can also regulate junctional proteins through the PI3-Kinase/Akt pathway [247]. Differently, glutamine would hamper the methotrexate-induced disruption of tight junction proteins through JNK and ERK [248]. In addition, AMPK could also be involved as a downstream target regulated by calcium/CaMKK2 signaling in response to glutamine supplementation [188].

Food-supplemented glutamine upregulated the HSP70 levels in peripheral blood mononuclear cells (PBMC) of human exercise-induced HS [243]. This can result in a reduction of pro-inflammatory cytokine secretion and thus an increased anti-inflammatory capacity and prevention of intestinal integrity disruption [243]. Another human study has supported the role of glutamine in reducing pro-inflammatory cytokine secretion, such as IL-6 and IL-8, while increasing the anti-inflammatory cytokine IL-10 in the exercise-induced “leaky” intestines [249] Moreover, feed supplementation with glutamine is associated with a reduction in LPS-induced intestinal inflammation in infant rats [250]. A review summarized that glutamine can reduce inflammatory responses observed in different animal IBD models [251]. Calves provided with alanyl-glutamine displayed an improvement in gain performance and health status concurrent with increases in blood CD2+ and CD4+ lymphocytes, the ratio of CD4+/CD8+, serum IgA and IgG, intestinal mucosal s-IgA, while decreasing the occurrence of diarrhea [252]. Dietary glutamine supplementation decreased TNF-α levels, D-lactate, serum diamine oxidase (DAO) activity and soluble intercellular adhesion molecule (sICAM)-1 concentration, and increased IL-10 levels in the intestinal mucosa of broilers [253]. The signaling pathway by which glutamine protects against inflammatory conditions is at least in part through stimulation of IκBα by HSP70 and associated suppression of NF-κB cascade [243,254].

In conclusion, amino acids like arginine and glutamine play an important role in protein biosynthesis, but also exert physiological effects on signal transduction pathways that regulate immunity, preserve epithelial integrity, and regulate antioxidation and energy metabolism. Beneficial effects of arginine and glutamine in experimental models of intestinal disorders have been frequently reported. Despite promising data in experimental models, further studies are needed to evaluate amino acid supplementation in clinical practice. The effects of arginine and glutamine on intestinal integrity and immunomodulation are summarized in Table 4.

## 4. Concluding Remarks

**Figure 3:**
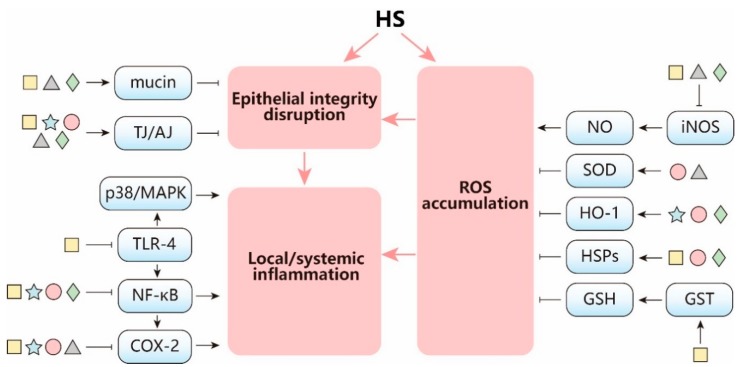
The pathways behind the protective effects of nutritional supplementation on HS-induced intestinal integrity disruption, inflammation, and oxidative stress. Pre-/probiotics (yellow square ⬜), α-lipoic acid (blue star ⭐), resveratrol (red circle ⚪), DHA/EPA (grey triangle 🔺) and amino acids (green diamond 🔷) not only restore intestinal epithelial integrity by increasing TJ/AJ protein and mucin expression, but also participate in anti-inflammation and stress resilience pathways. Normal arrow: enhancement or activation; bar-headed arrow: reduction or inhibition.

HS is considered as an important environmental stressor that is of increasing public health concern. Intervention strategies that can prevent, control, and reduce the pathologies (and even mortality) due to HS in humans and animals are therefore gaining increasing attention. Accumulating evidence suggests that the disruption of intestinal integrity followed by a generalized inflammatory response is a key event in human and animal pathologies under HS conditions. Subsequently, an increasing number of studies focus on the understanding of the molecular mechanisms involved in HS-induced inflammation and intestinal barrier disruption with the aim to introduce efficient strategies to preserve the physiologic performance of the gut (Figure 3). Future research needs to focus on the cellular and molecular pathways that act behind hyperthermia and hypoxia-induced pathologies. Currently, only a few classic cellular mechanisms, such as the heat shock and oxidative stress response, are clearly described. Furthermore, there are several gaps within the existing knowledge related to the effect of HS on junctional proteins.

More than 40 tight junction proteins and more than 20 cadherin proteins have been identified, yet none of the junctional proteins studied have been found to be exclusively responsible for barrier homeostasis under hyperthermia or hypoxia conditions. It has been documented that HSP can bind to TJ proteins, such as ZO-1 and occludin, however, the causality between upregulation of specific HSP and restoration of TJ proteins still needs to be clarified. In addition, apart from a study by Bidmon-Fliegenschnee et al. [39], and some “non-intestinal” studies suggesting a link between HSP/HSF and the catenin/cadherin family [255,256], our understanding about the interaction of HSP and AJ proteins is limited.

As depicted in Figure 3, the described nutritional substances have a broad range of effects on HS-induced intestinal integrity disruption, inflammation, and oxidative stress. Nutritional substances, which have the potency to preserve not only cellular homeostasis by enhancing non-specific cellular defense systems, but also maintain intestinal integrity, are considered as promising feed/food supplements to protect animals and humans against the adverse effects of HS.

Accumulating evidence indicates that provision of a combination of nutritional substances is more effective than treatment with a single dietary component. Synbiotics, a combination of probiotics and prebiotics, have been shown to exert synergistic effects in the intestinal tract [62,257]. Microbiota-stabilizing compounds in combination with other nutritional substances can also enhance beneficial health effects and can possibly be used in a broader range of clinical conditions. For example, a prebiotic fiber diet combining with resveratrol and DHA was effective in lessening brain injury in rats [258]. It will be a promising approach in the future to investigate the combination of different nutritional substances in (HS-associated) intestinal problems, offering innumerable possibilities to therapeutic practice.

## Figures and Tables

**Table 1:** The effects of pro-/prebiotics on intestinal integrity and immunomodulation.

Name		Integrity	Immunomodulation	Other Effect(s)	Reference
**Pro-biotics**	*Lactobacillus* species	TEER↑	IL-10↑	Integrin-p38 MAPK activation↑	[77,82,83,84,88,89,135]
		Intestinal permeability↓	IL-27↑	HSP expression↑	
		ZO-1↑	IL-1↓	Antioxidative capacity↑	
		occludin↑	IL-6↓	Nutrient transporters↑	
		E-cadherin↑	TNF-α↓		
		claudin-2↑	NF-κB activation↓		
	*Bifidobacterium* species	claudin-3↑	Corticosterone↓	Mucin genes transcription and protein production↑	[77,88,89]
		Morphological damage↓	IgA secreting cells↑		
		β-catenin↑	Intraepithelial lymphocytes↓		
	*Bacillus* species				[79,80,85,88,89]
	*E. coli* Nissle	ZO-2 dissociation↓		-	[81,88,89]
	*Streptococcus thermophiles*	occludin delocalization↓		-	[84,88,89]
	HMO	ZO-1↑	IL-10↑	Mucus production↑	[109,123,130,136]
		occludin↑	TLR-4↓	HIF-1α↓	
		JAM-A↑	NF-κB translocation↓	Cleaved caspase-3↓	
		Crypt proliferation↑	p38 MAPK activation↓	EGFR activation↑	
		Intestinal permeability↓			
**Pre-biotics**	GOS	TEER↑	IL-6 mRNA↓	HSP expression↓	[99,100,102,103,119]
		Intestinal permeability↓	IL-8 mRNA↓	Populations of probiotics↑	
		occludin↑	TLR-4↓	HO-1 expression↓	
		claudin-3↑	IL-33↓		
		E-cadherin↑	CXCL-8↓		
			CXCL-1↓		
			CXCL-2↓		
	MOS	Intestinal permeability↓	-	Goblet cells↑	[106,107,108]
		permeability↓		Populations of probiotics↑	
		Villus height↑		*E. coli* load↑	
	COS	Intestinal permeability↓	-	-	[106,107]
		Morphological damage↓			
	FOS	TEER↑		Colonic SCFA concentration↑	[110,115,137]
		Intestinal permeability↓	-	Mucosal damage↓	
		occludin↑			
		ZO-1↑			
	Chitosan oligosaccharides	TEER↑	IL-6↓	GST↑	[111,119,132,133]
		Epithelial degeneration↓	TNF-α↓		
		TJ proteins redistribution and distortion↓	COX-2 activation↓		
			iNOS↓		
			NO production↓		
			NF-κB translocation↓		

Upwards arrow: Increase or enhancement; downwards arrow: Decrease or inhibition. TEER: trans-epithelial electrical resistance; IL: interleukin; TNF: tumor necrosis factor; NF-κB: nuclear factor κ-light-chain-enhancer of activated B cells; IgA: immunoglobulin A; MAPK: mitogen-activated protein kinase; JAM: junctional adhesion molecule; TLR: toll-like receptor; HMO: human milk oligosaccharides; HIF: hypoxia-inducible factor; EGFR: epidermal growth factor receptor; GOS: galacto-oligosaccharides; CXCL: C-X-C motif chemokine ligand; HO-1: haem oxygenase 1; MOS: mannan-oligosaccharides; COS: cello-oligosaccharides; FOS: fructo-oligosaccharides; SCFA: short chain fatty acids; GST: glutathione S-transferase; COX: cyclooxygenase; iNOS: inducible isoform of NOS; NO: nitric oxide.

**Table 2:** The effects of α-lipoic acid and resveratrol on intestinal integrity and immunomodulation.

Compound	Integrity	Immunomodulation	Other Effect(s)	Reference
**α-lipoic acid**	Intestinal permeability↓	COX-2 activation↓	Epithelial proliferation↑	[144,145,146,147,148,150,151,152,153,154]
	ZO-1↑	IL-17↓	HSP70 expression↑	
	occludin↑	IL-6↓	HO-1 activation↑	
	E-cadherin↑	TNF-α↓		
	Morphological damage↓	IκB activation↑		
**Resveratrol**	ZO-1↑	IL-6 mRNA↓	MDA↓	[155,156,160,161,162,163,165,168,169]
	occludin↑	IL-1β mRNA↓	SOD↑	
	TEER↑	PTGS1 mRNA↓	GSH↓	
	Intestinal permeability↓	COX-2 activation↓	ROS↓	
	claudin-1↑	NF-κB activation↓	HO-1 activation↑	
	claudin-4↑		HSP70↑	
	Crypt depth↓		HSP90↑	
	Villus height↑			

Upwards arrow: Increase or enhancement; downwards arrow: Decrease or inhibition. PTGS1: prostaglandin G/H synthase 1; MDA: malondialdehyde; SOD: superoxide dismutase; GSH: glutathione; ROS: reactive oxygen species.

**Table 3:** The effects of polyunsaturated fatty acids (PUFA) on intestinal integrity and immunomodulation.

Compound	Integrity	Immunomodulation	Other Effect(s)	Reference
**EPA and DHA**	TEER↑	Acute inflammation↓	Mucosal damage↓	[172,174,175,176,177,179,180,181,182]
	Intestinal permeability↓	IL-1β↓	ROS production↓	
	occludin↑	IL-6↓	SOD↑	
	ZO-1↑	IL-17↓	CAT↑	
	E-cadherin↑	TNF-α↓	Total nitrate/nitrite ratio↓	
	TJ proteins redistribution and distortion↓	INF-γ↓	Microbiota composition restore↑	
		COX-2 activation↓	MUC-2 gene↑	
		iNOS↓	Cytokeratin gene↑	
		cGMP↓		

Upwards arrow: Increase or enhancement; downwards arrow: Decrease or inhibition. EPA: eicosapentaenoic acid; DHA: docosahexaenoic acid; CAT: catalase; cGMP: cyclic guanosine monophosphate.

**Table 4:** The effects of arginine and glutamine on intestinal integrity and immunomodulation.

Compound	Integrity	Immunomodulation	Other Effect(s)	Reference
**Arginine**	Intestinal permeability↓	iNOS↑	Intestinal necrosis↓	[35,207,208,209,218,223,224]
	TEER↑	Intestinal s-IgA↑	Mucus production and fluid secretion↑	
	ZO-1↑	NF-κB activation↓		
	E-cadherin↑	Pro-inflammatory cytokines↓		
		pro-inflammatory chemokines↓		
	Villus height↑			
**Glutamine**	Intestinal permeability↓	NF-κB activation↑	Mucus production↑	[188,235,236,237,238,239,240,241,242,243,245,246,249,250,251,252,253,254]
	Villus atrophy↓	CD2+ and CD4+ lymphocytes↑	HSP70 expression↑	
		CD4+/CD8+↑	HSF-1 expression↑	
	occludin↑	Serum IgA and IgG↑	HO-1 expression↑	
	claudin-1↑	Intestinal mucosal s-IgA↑	Cell viability and antioxidant capacity↑	
	claudin-4↑	TNF-α↓	Hyperthermia↓	
	JAM-A↑	D-lactate↓	Diarrhea occurrence↓	
	ZO-1, ZO-2 and ZO-3↑	DAO activity↓		
	E-cadherin↑	sICAM-1↓		
	β-catenin↑	IL-6↓		
		IL-8↓		
		IL-10↑		

Upwards arrow: Increase or enhancement; downwards arrow: Decrease or inhibition. IgG: immunoglobulin G; DAO: diamine oxidase; sICAM: soluble intercellular adhesion molecule.
